# The burden and correlates of multiple cardiometabolic risk factors in a semi-urban population of Nepal: a community-based cross-sectional study

**DOI:** 10.1038/s41598-019-51454-9

**Published:** 2019-10-25

**Authors:** Bishal Gyawali, Shiva Raj Mishra, Saruna Ghimire, Martin Rune Hassan Hansen, Kishor Jung Shah, Koshal Chandra Subedee, Pabitra Babu Soti, Dinesh Neupane, Per Kallestrup

**Affiliations:** 1 Community Health Development Nepal (CHEDEN), Kathmandu, Nepal; 2 Global Health Section, Department of Public Health, University of Copenhagen, Copenhagen, Denmark; 3 Nepal Development Society, Bharatpur, Nepal; 4 Department of Sociology and Gerontology, Miami University, Oxford, OH United States of America; 5 Department of Public Health, Aarhus University, Aarhus, Denmark; 6 National Research Centre for the Working Environment, Copenhagen, Denmark; 7 Department of Epidemiology, Welch Center for Prevention, Epidemiology, and Clinical Research Johns Hopkins Bloomberg School of Public Health, Baltimore, United States of America

**Keywords:** Public health, Cardiovascular diseases

## Abstract

This study assessed the burden and correlates of three cardiometabolic risk factors, (hypertension, diabetes, and overweight/obesity), and their possible clustering patterns in a semi-urban population of Nepal. Data were obtained from a community-based management of non-communicable disease in Nepal (COBIN) Wave II study, which included 2,310 adults aged 25–64 years in a semi-urban area of Pokhara Metropolitan City of Nepal, using the World Health Organization-STEPS questionnaire. Unadjusted and adjusted binary logistic regression models were used to study the correlates of the individual risk factors and their clustering. The prevalence of hypertension, diabetes, and overweight/obesity was 34.5%, 11.7%, and 52.9%, respectively. In total, 68.2% of the participants had at least one risk factor and many participants had two risks in combination: 6.8% for ‘hypertension and diabetes’, 7.4% for ‘diabetes and overweight/obesity’ and 21.4% for ‘hypertension and overweight/obesity’. In total, 4.7% had all three risk factors. Janajati ethnicity (1.4–2.1 times), male gender (1.5 times) and family history of diabetes (1.4–3.4 times) were associated with presence of individual risk factors. Similarly, Janajati ethnicity (aOR: 4.31, 95% CI: 2.53–7.32), current smoking (aOR: 4.81, 95% CI: 2.27–10.21), and family history of diabetes (aOR: 4.60, 95% CI: 2.67–7.91) were associated with presence of all three risk factors. Our study found a high prevalence of all single and combined cardiometabolic risk factors in Nepal. It underlines the need to manage risk factors in aggregate and plan prevention activities targeting multiple risk factors.

## Introduction

Cardiovascular disease (CVD), a leading cause of global morbidity and mortality, accounts for 17.9 million deaths worldwide annually^1^. In 2012, it was estimated that 7.4 million died due to coronary heart diseases and 6.7 million died due to stroke^2^. Over 75% of cardiovascular deaths take place in low-and middle-income countries (LMICs)^1^. Additionally, CVD contributes to the global economic burden by increasing health-care expenditures, lower productivity at work, increasing the number of sick days, causing permanent disability^3^. Nepal, a low-income country in South Asia, is experiencing a similar increasing trend in CVD morbidity and mortality. The mortality attributed to CVD in Nepal has increased rapidly from 22% to 25% between 2004 and 2008^4,5^.

The public health care spending in Nepal is still focused on infectious diseases, and the resources allocated to fighting CVD have not kept up with its increasing burden. As is common in poor resource settings, cardiometabolic risk factors at subclinical stages are often not presented to health professionals until some serious symptoms of major CVD arise^6^. The resulting late diagnosis precludes the benefits of early diagnosis and management of the conditions and prevention of complications. Further, although patients with multiple risk factors may have preventive and clinical needs that are different than those with a single factor, disease management protocols in Nepal primarily focus on the treatment of symptoms presented to the health professionals, and often competing risk factors are not handled.

There is a dearth of information on the major drivers of the concurrent cardiometabolic risk factors among the Nepalese population. A previous study evaluated the prevalence and concurrence of cardiometabolic risk factors but was limited to Eastern parts of the country^7^. A recent study evaluated the prevalence and determinants of metabolic syndrome among nationally representative Nepalese adults, however, failed to include some important socio-demographic determinants of CVD, such as monthly income^8^. To our knowledge, no studies in Nepal have adequately evaluated socio-demographic and lifestyle correlates of the prevalence of cardiometabolic risk profiles, individually as well as in combination as multiple cardiometabolic risk factors. The current study aimed to assess the burden and correlates of three cardiometabolic risk factors (hypertension, diabetes, and overweight/obesity), and their clustering patterns in a semi-urban population of Nepal.

## Results

### Socio-demographics and behavioural characteristics

Table 1 provides the socio-demographic and behavioural characteristics of the participants and has been previously reported^9^. Three-fifth of the participants were middle-aged (35–54 years). The majority of the participants were female (68%), Upper caste (54%), and had an average monthly income of 20,000 Nepali Rupees (NPR) or greater (65%).

**Table 1:** Socio-demographic and behavioural characteristics of the study participants.

Characteristics	n (%)
**Age group** (**years**)	
25–34	288 (13)
35–44	676 (29)
45–54	727 (31)
55–64	619 (27)
**Gender**	
Male	736 (32)
Female	1,574 (68)
**Ethnicity**	
Upper caste	1,254 (54)
Janajatis	742 (32)
Dalits and ethnic minorities	314 (14)
**Monthly income** (**NPR**)^*^	
<20,000	817 (35)
≥20,000	1,493 (65)
**Current smoking**	
Yes	365 (16)
No	1,945 (84)
**Harmful alcohol use**	
Yes	307 (13)
No	2,003 (87)
**Physical activity**	
Low	264 (11)
High	2,046 (89)
**Servings of fruits/vegetables**	
≥5 servings	122 (5)
<5 servings	2,188 (95)
**History of heart diseases**	
Yes	75 (3)
No	2,235 (97)
**Family history of diabetes**	
Yes	455 (20)
No	1,855 (80)
**Diabetes**	
Yes	271 (12)
No	2,039 (88)
**Overweight/obesity**	
Yes	1,222 (53)
No	1,088 (47)
**Hypertension**	
Yes	797 (35)
No	1,513 (65)

Socio-demographic and behavioural characteristics of the study participants (N = 2,310). *100 NPR ~ 1 US dollar.

A sizeable proportion of the participants smoked daily (16%) or used alcohol at harmful level (13%). Most of the participants displayed a high level of physical activity (89%) but poor fruit/vegetable intake; 95% did not consume the recommended five or more servings per week. One-fifth of the participants had a family history of diabetes.

### Prevalence and correlates of cardiometabolic risk factors

More than 68% of the participants had at least one cardiometabolic risk factor. The prevalence of hypertension was 34.5%, diabetes was 11.7%, and overweight/obesity was 52.9%. Supplemental Table 1 shows the prevalence of the three risk factors by demographic and behavioural characteristics. The prevalence of diabetes and hypertension, showed an increasing trend across the age categories, whereas a peak in the prevalence of overweight/obesity was observed for the age group 35–54 years. The prevalence of diabetes, hypertension, and overweight/obesity, individually, was higher in females, Upper caste, and those with higher income (Supplemental Table 1).

**Figure 1:**
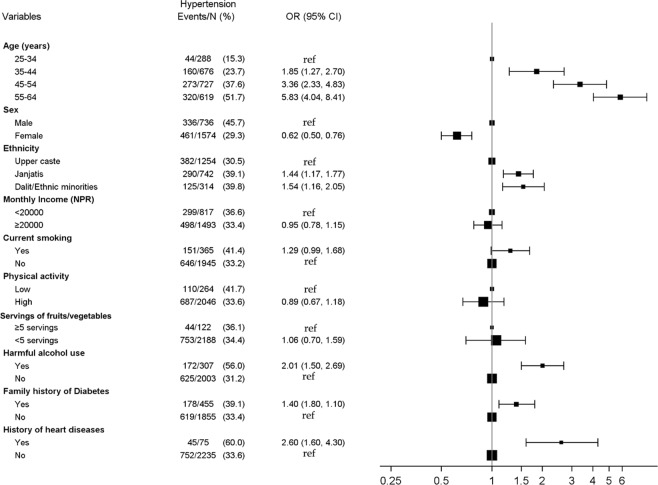
Risk factors for hypertension.

**Figure 2:**
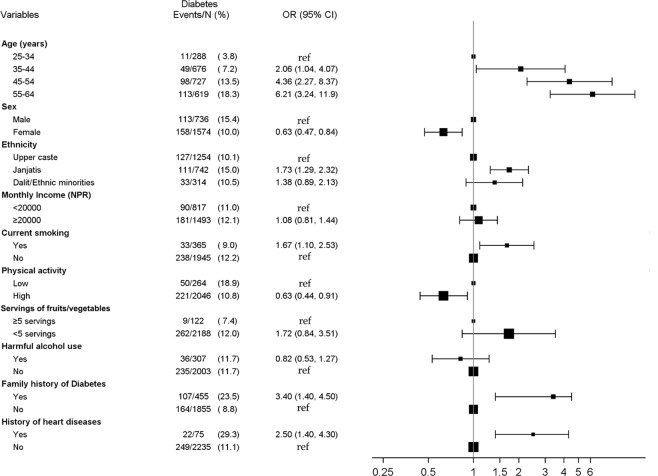
Risk factors for diabetes.

**Figure 3:**
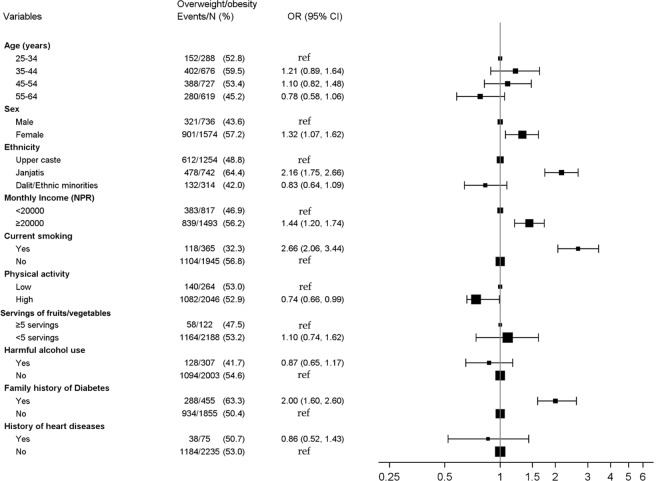
Risk factors for overweight/obesity.

In multivariate logistic regression analyses, hypertension and diabetes showed a positive dose-response relationship with the age category. Compared to males, females were less likely to be hypertensive (aOR: 0.62, 95% CI: 0.50–0.76) and diabetic (aOR: 0.63, 95% CI: 0.47–0.84) but more likely to be overweight/obese (aOR: 1.32, 95% CI: 1.07–1.62). Compared to the Upper caste, Janajati ethnicity had higher odds of being diabetic (aOR: 1.73, 95% CI: 1.29–2.32) and overweight/obese (aOR: 2.16, 95% CI: 1.75–2.66). Higher odds of overweight/obesity were seen for those with higher income (aOR 1.44, 95% CI 1.20–1.74), compared to those with lower income. Current smokers had higher odds of two risk factors, i.e., diabetes (aOR: 1.67, 95% CI: 1.10–2.53) and overweight/obesity (aOR: 2.66, 95% CI: 2.06–3.44). Higher physical activity was associated with reduced risk of diabetes (aOR: 0.63, 95% CI: 0.44–0.91) and overweight/obesity (aOR: 0.74, 95% CI: 0.66–0.99), and harmful use of alcohol was associated with increased odds of hypertension (aOR: 2.01, 95% CI: 1.50–2.69). A family history of diabetes was associated with increased odds of each of the three-individual risk factor whereas participants’ history of heart disease was associated with increased odds of hypertension and diabetes. Risk factors for hypertension, diabetes and overweight/obesity are shown in Figs 1–3.

### Two-way clustering: prevalence and correlates

A sizable proportion of the participants showed two-way unique combinations of risk factors; the prevalence ranging from 6.8% to 66.0% (hypertension and diabetes: 6.8%, hypertension or diabetes: 39.4%, hypertension and overweight/obesity: 21.4%, hypertension or overweight/obesity: 66.0%, diabetes and overweight/obesity: 7.4%; diabetes or overweight/obesity: 57.2%). Supplemental Table 1 shows the prevalence of the six-different two-way clustering of risk factors by demographic and behavioral characteristics. The prevalence of the two-way clustering of risk factors was higher in older age groups, female, Upper caste, those with higher monthly income (≥20,000 NPR), and those having a history of heart diseases Supplemental Table 1.

In multivariate analyses (Table 2), the odds of any two-risk factors clustering showed a gradually increasing trend by age categories, except for the co-occurrence of diabetes and overweight/obesity where no significant association was reported across the higher age categories. Likewise, participants from the Janajati ethnicity and having a family history of diabetes were more likely to have any two-risk factors clustering compared to their respective counterparts. Female gender and history of heart disease were associated with lower odds of having either hypertension or diabetes or both. Smoking was associated with higher odds of having all six two-way unique combinations of risk factors (Table 2).

**Table 2:** Two-way clustering of cardiometabolic risk factors by demographic and behavioural characteristics.

Demographic and behavioral characteristics	Hypertension and diabetes	Hypertension or diabetes	Hypertension and overweight/obesity	Hypertension or overweight/obesity	Diabetes and overweight/obesity	Diabetes or overweight/obesity
	OR (95% CI)	OR (95% CI)	OR (95% CI)	OR (95% CI)	OR (95% CI)	OR (95% CI)
	Adjusted	Adjusted	Adjusted	Adjusted	Adjusted	Adjusted
**Age group**						
25–34	Ref	Ref	Ref	Ref	Ref	Ref
35–44	**4**.**86** (**1**.**10–21**.**46**)	**1**.**89** (**1**.**33–2**.**69**)	**2**.**07** (**1**.**29–3**.**31**)	**1**.**54** (**1**.**15–2**.**06**)	**3**.**10** (**1**.**12–8**.**56**)	**1**.**36** (**1**.**01–1**.**82**)
45–54	**17**.**06** (**4**.**06–71**.**60**)	**3**.**56** (**2**.**52–5**.**01**)	**3**.**10** (**1**.**97–4**.**87**)	**1**.**62** (**1**.**21–2**.**16**)	**8**.**02** (**3**.**02–21**.**29**)	1.23 (0.92–1.64)
55–64	**42**.**02** (**10**.**05–175**.**70**)	**5**.**92** (**4**.**17–8**.**40**)	**3**.**95** (**2**.**50–6**.**26**)	**1**.**85** (**1**.**37–2**.**50**)	**9**.**66** (**3**.**64–25**.**69**)	1.04 (0.77–1.40)
**Sex**						
Male	Ref	Ref	Ref	Ref	Ref	**Ref**
Female	**0**.**45** (**0**.**30–0**.**67**)	**0**.**59** (**0**.**48–0**.**73**)	0.87 (0.66–1.15)	1.12 (0.91–1.38)	0.75 (0.50–1.12)	**1**.**38** (**1**.**13–1**.**70**)
**Ethnicity**						
Upper caste	Ref	Ref	Ref	Ref	Ref	**Ref**
Janajatis	**2**.**19** (**1**.**46–3**.**28**)	**1**.**48** (**1**.**20–1**.**81**)	**2**.**59** (**1**.**97–3**.**41**)	**1**.**85** (**1**.**50–2**.**28**)	**3**.**51** (**2**.**35–5**.**26**)	**2**.**10** (**1**.**72–2**.**58**)
Dalit/ethnic minorities	1.64 (0.87–3.10)	**1**.**66** (**1**.**25–2**.**19**)	1.28 (0.88–1.86)	1.06 (0.81–1.39)	1.63 (0.93–2.86)	0.85 (0.65–1.11)
**Monthly income** (**NPR**)						
<20000	Ref	Ref	Ref	**Ref**	Ref	Ref
≥20000	1.18 (0.79–1.76)	0.92 (0.76–1.11)	1.11 (0.86–1.42)	**1**.**24** (**1**.**03–1**.**49**)	1.35 (0.92–1.10)	**1**.**30** (**1**.**09–1**.**56**)
**Current smoking**						
No	Ref	Ref	Ref	Ref	Ref	Ref
Yes	**2**.**45** (**1**.**39–4**.**32**)	**1**.**33** (**1**.**02–1**.**73**)	**2**.**57** (**1**.**78–3**.**70**)	**1**.**80** (**1**.**39–2**.**32**)	**3**.**11** (**1**.**77–5**.**50**)	**2**.**28** (**1**.**76–2**.**95**)
**Physical activity**						
Low	Ref	Ref	Ref	Ref	Ref	Ref
High	0.66 (0.40–1.09)	0.83 (0.63–1.10)	0.79 (0.54–1.15)	0.82 (0.62–1.10)	0.68 (0.41–1.15)	0.76 (0.58–1.01)
**Servings of fruits/vegetables**						
≥5 servings	Ref	Ref	Ref	Ref	Ref	Ref
<5 servings	2.20 (0.75–6.45)	1.10 (0.74–1.65)	1.07 (0.64–1.78)	1.24 (0.84–1.82)	1.59 (0.65–3.89)	1.19 (0.81–1.75)
**Harmful alcohol use**						
No	Ref	Ref	**Ref**	Ref	Ref	Ref
Yes	1.65 (0.94–2.90)	**1**.**75** (**1**.**31–2**.**35**)	**1**.**63** (**1**.**11–2**.**40**)	**1**.**43** (**1**.**05–1**.**95**)	0.72 (0.40–1.31)	0.93 (0.69–1.24)
**Family history of diabetes**						
No	Ref	Ref	Ref	Ref	Ref	Ref
Yes	**3**.**57** (**2**.**35–5**.**40**)	**1**.**97** (**1**.**57–2**.**46**)	**1**.**91** (**1**.**41–2**.**59**)	**1**.**70** (**1**.**34–2**.**19**)	**5**.**16** (**3**.**48–7**.**65**)	**1**.**99** (**1**.**58–2**.**50**)
**History of heart diseases**						
No	Ref	Ref	Ref	Ref	Ref	Ref
Yes	**4**.**65** (**2**.**19–9**.**87**)	**2**.**76** (**1**.**64–4**.**62**)	**2**.**32** (**1**.**22–4**.**39**)	1.44 (0.84–2.48)	1.45 (0.58–3.62)	1.5 (0.92–2.60)

Adjusted for all demographic and behavioral characteristics given in the table. Unadjusted estimates are provided in Supplemental Table 2. *100 NPR ~1 US dollar. Significant estimates are bolded.

### Three-way clustering: prevalence and correlates

A substantial proportion of study participants had at least one (68.2%) or all three (4.7%) cardiometabolic risk factors. Supplemental Table 1 shows the prevalence of the three-way clustering of the risk factors by demographic and behavioral characteristics. Among those with at least one of the risk factors, the prevalence of clustering was inverted J-shape with the highest prevalence (32%) observed for the age group 45–54 years. Within-group comparison suggest that prevalence of at least one cardiometabolic risk factor was highest for female (69%), Upper caste ethnic group (51%), and those with higher income (66%) compared to their respective counterparts (Supplemental Table 1). Likewise, the prevalence of all three cardiometabolic risk factors was higher in women (53%) compared to men (47%), and it increased linearly with age groups (Supplemental Table 1).

In multivariate analyses, the two types of three-way clustering pattern (at least one risk factors and all three risk factors), showed a gradual increase in the odds of risk factors with age categories, but the magnitude of the odds ratio was much higher for all three risk factors. Janajati ethnicity, current smoking, and family history of diabetes was associated with higher odds of having both all three and at least one risk factor. Females had lower odds (aOR: 0.48, 95% CI: 0.29–0.80) of having all three risk factors compared to men (Table 3).

**Table 3:** Three-way clustering of cardiometabolic risk factors by demographic and behavioural characteristics.

Demographic and behavioural characteristics	At least one risk factor (n = 1,575, 68.2%) (Diabetes or hypertension or overweight/obesity) OR (95% CI)		All three risk factors (n = 108, 4.7%) (Diabetes and hypertension and overweight/obesity) OR (95% CI)	
	Unadjusted	Adjusted	Unadjusted	Adjusted
**Age group**				
25–34	Ref	Ref	Ref	Ref
35–44	**1**.**54** (**1**.**16–2**.**05**)	**1**.**56** (**1**.**16–2**.**09**)	5.61 (0.71–44.39)	6.66 (0.82–54.33)
45–54	**1**.**56** (**1**.**17–2**.**07**)	**1**.**67** (**1**.**25–2**.**24**)	**21**.**06** (**2**.**86–155**.**11**)	**26**.**71** (**3**.**52–202**.**80**)
55–64	**1**.**74** (**1**.**30–2**.**34**)	**1**.**96** (**1**.**44–2**.**66**)	**38**.**11** (**5**.**21–278**.**95**)	**59**.**61** (**7**.**85–452**.**42**)
**Sex**				
Male	Ref	Ref	Ref	Ref
Female	1.07 (0.89–1.30)	1.07 (0.86–1.32)	**0**.**55** (**0**.**36–0**.**82**)	**0**.**48** (**0**.**29–0**.**80**)
**Ethnicity**				
Upper caste	Ref	Ref	Ref	Ref
Janajatis	**1**.**82** (**1**.**48–2**.**24**)	**1**.**87** (**1**.**51–2**.**32**)	**3**.**71** (**2**.**38–5**.**78**)	**4**.**31** (**2**.**53–7**.**32**)
Dalit/ethnic minorities	1.01 (0.78–1.30)	1.10 (0.84–1.45)	1.34 (0.69–2.59)	2.02 (0.96–4.26)
**Monthly income** (**NPR**)				
<20,000	Ref	Ref	Ref	Ref
≥20,000	**1**.**23** (**1**.**03–1**.**48**)	1.18 (0.98–1.43)	1.43 (0.93–2.21)	1.24 (0.75–2.05)
**Current smoking**				
No	Ref	Ref	Ref	Ref
Yes	**1**.**64** (**1**.**31–2**.**07**)	**1**.**85** (**1**.**43–2**.**41**)	**2**.**53** (**1**.**29–4**.**98**)	**4**.**81** (**2**.**27–10**.**21**)
**Physical activity**				
Low	Ref	Ref	Ref	Ref
High	0.77 (0.57–1.02)	0.78 (0.58–1.06)	**0**.**50** (**0**.**29–0**.**87**)	0.73 (0.38–1.43)
**Servings of fruits/vegetables**				
≥5 servings	Ref	Ref	Ref	Ref
<5 servings	1.32 (0.90–1.92)	1.28 (0.87–1.89)	2.34 (0.71–7.65)	2.19 (0.63–7.66)
**Harmful alcohol use**				
No	Ref	Ref	Ref	Ref
Yes	1.12 (0.87–1.46)	1.35 (0.98–1.84)	1.32 (0.75–2.32)	1.03 (0.49–2.15)
**Family history of diabetes**				
No	Ref	Ref	Ref	Ref
Yes	**1**.**96** (**1**.**53–2**.**50**)	**1**.**98** (**1**.**54–2**.**55**)	**4**.**40** (**2**.**83–6**.**84**)	**4**.**60** (**2**.**67–7**.**91**)
**History of heart diseases**				
No	Ref	Ref	Ref	Ref
Yes	**1**.**90** (**1**.**07–3**.**37**)	1.76 (0.98–3.18)	**4**.**36** (**1**.**86–10**.**23**)	2.89 (0.96–8.71)

Adjusted for all demographic and behavioral characteristics given in the table. *100 NPR ~1 US dollar. Significant estimates are bolded.

## Discussion

With an aim to assess the burden and correlates of three cardiometabolic risk factors, i.e., hypertension, diabetes and overweight/obesity, and their possible clustering patterns in a semi-urban population of Nepal, we found evidence of a high burden of the risk factors, individually and as clusters. In fact, 68.2% of the participants had at least one cardiometabolic risk factor, and 4.7% had all three risk factors. The risk factors, individually and clusters, showed increasing odds with age, male gender, and Janajati ethnicity. Similarly, the participant’s family history of diabetes was associated with higher odds of any single or combined risk factors.

The high burden of hypertension (34.5%), overweight/obesity (52.9%), diabetes (11.7%), and their clustering observed in this study is concerning, although not surprising. Compared to the previous study from eastern Nepal, the prevalence of overweight/obesity (60.7%) was slightly higher, but previous estimates for hypertension (33.9%) and diabetes (6.3%) were slightly lower than ours^7^. The urban-rural disparity in the distribution of cardiometabolic risk factors, which is well documented in the Nepalese context^10^ as well as globally^11,12^ may explain the discrepancy in findings. Lifestyle changes, specifically access to heart non-healthy dietary choices and sedentary activities, as a consequence of rapid urbanization has particularly localized these risk factors to urban areas. The semi-urban locality of our study setting suggests our estimates to be in-between those from the rural and urban area.

The risk factors, individually, and as a cluster, showed increasing odds with age, male gender and Janajati ethnicity in our study which is consistent with the previous findings from Nepal^7,10^ and globally^13^. The decline in cardiovascular health with aging was expected given that with increasing age, there is a gradual decrease in physiological functions, increased vulnerability to diseases, and a general decline in the capacity of the individual^14^. More importantly, the magnitude of the odds ratio increased gradually from a single risk factor to two-way clustering and reached maximum for three-way clustering. Older population exhibits a naturally higher burden of comorbidities due to biological aging^15^, often exacerbated by the frequent clustering of risk factors in older adults^16^. It also hints towards the accumulation of risk factors over the life course, highlighting a longitudinal growth in CVD risk. Future studies with longitudinal designs have the opportunity to study pathways leading to risk factors clustering.

The findings on increased odds of risk factors for participants of Janajati ethnicity was interesting, but also not surprising. Based on historical association with the caste system, persistent in driving disparities between ethnic groups, three major ethnic groups are identified: Upper caste, Janajatis, and Dalits. These three ethnic groups would be expected to generally represent higher, medium, and lower social status, with the Dalit representing the most marginalized of all groups^17^. The government of Nepal has recognized Janajati and Dalit ethnic groups as marginalized groups and special provisions are in place to provide them with an equal opportunity for health, education, public jobs, and etc.^17^ Although, Janajatis are generally considered less privileged, our participants do not fall into that category. Our Janajati participants are likely to have better socio-economic status, sedentary and unhealthy lifestyle because most of them are active in duty or retired Gurkha army personnel, employed in the workforce by the governments of Nepal, India, and Britain. A previous study has reported heavy alcohol use, tobacco smoking and sedentary lifestyle among Gurkha army personnel^18^, which constitutes cardiometabolic risk factors. As a consequence, high prevalence of CVDs has been reported among people of Janajati ethnicity in Nepal^19,20^. The odds ratio for Janajatis are adjusted for the other risk factors in the study, but an association between Janajatis and CVD risk persists, likely because other unmeasured risk factors, such as total caloric intake and genetic factors also differ between the ethnic groups. Including the ethnic group in the analysis is thus also a way of handling unmeasured confounders.

Likewise, current smoking and harmful alcohol consumption were associated with cardiometabolic risk factors in our study, not surprisingly since they are well established modifiable factors in cardiology^21–23^. Lifestyle choices are important contributors to the global rise in chronic disease, including CVDs^24^. Moreover, having a family history of diabetes was significantly associated with cardiometabolic risk factors in our study. Our findings are in line with most of the earlier studies, which demonstrated familial aggregation of diabetes and related phenotypes^25^.

Our finding that about two-thirds of the participants had at least one of the risk factors particularly concerns because risk factors together show a multiplicative, rather than merely additive, synergistic effect^26^. The high burden of risk factor clustering, on the one hand, supports the 2016 estimates of the Global Burden of Disease (GBD) for Nepal which established CVD as the leading cause of both mortality and morbidity^27^. On the other hand, it portends escalating rates of CVD-related morbidity and premature mortality in Nepal, and thus a need to curb these risk factors at the earliest onset. From policy and practice perspectives, the clustering highlights the maturation of cardiometabolic syndemics from single to multiple cardiometabolic risk factors in Nepal and posits a need to address these risk factors holistically in both policy and clinical practice.

### Strengths and limitation

Our study adds to the limited literature on cardiometabolic risk factors and their clustering in Nepal’s local context. Further, both previous studies evaluated the composite metabolic syndrome as the outcome and as such this is the first study to assess the burden of possible combinations of risk factors comprehensively. Our study has also benefited from a large and representative sample size and use of standardized STEPS tools.

This study also has some limitations. First, due to financial constraints, the measurement of cholesterol and lipid profiles, important markers of cardiometabolic risk, was not feasible. Likewise, measurement of glycosylated haemoglobin A (HbA1c) or oral glucose tolerance tests was not possible. The latter limitation is somehow eased since, according to WHO guidelines, a single fasting plasma glucose estimation is acceptable for epidemiological purposes^28^. Additional limitation includes the study participants were nested to COBIN cohort and some of them were part of intervention study that was conducted earlier. Further, cross-sectional study design, which although appropriate for the prevalence estimates of the risk factors, inhibits us from establishing any causal inference between correlates and outcomes. Future studies with prospective design may be helpful to elucidate the longitudinal progression of the cardiometabolic risk factors, from single to consecutive concurrence.

## Conclusions

Our study found a high prevalence and clustering of multiple cardiometabolic risk factors among adults in semi-urban Nepal, highlighting the growing cardiometabolic syndemics from single to multiple risk factors. The concentration of the risk factors, individually and as clusters, in specific groups such as older population, male, Janajati ethnicity, and those with a family history of diabetes posit specific higher risk groups for targeting interventions. Our findings of high burden and clustering of cardiometabolic risk factors coupled with recent GBD estimate establishing CVD as the leading cause of both mortality and morbidity in Nepal, make it clear that prevention, treatment, and control of cardiometabolic risk factors should be a public health priority in the country. Interventions tailored to reduce the burden of multiple risk factors before cardiometabolic diseases appear, such as raising awareness and conducting regular health screenings are urgently needed in a resource constrained setting like Nepal.

## Methods

### Study design and population

This study is embedded within a community-based management of non-communicable disease in Nepal study (COBIN)^29^. The COBIN, is an ongoing prospective cohort that aims to examine non communicable disease burden and identifying possible strategies for community-based management of major non-communicable diseases. The COBIN cohort was established in 2015 (COBIN Wave I) by selecting 2,815 random participants from a semi-urban area of Pokhara Metropolitan City of Nepal. We did a follow up study (COBIN Wave II) after one year among 2,310 participants (82%)^30^. At follow up, we added additional information on fasting blood glucose test. Ethical Review Board of Nepal Health Research Council approved this study (reference number 766; reg. no 263/2016). Respondents provided informed written consent before data collection was initiated. Participation was voluntary, and participants’ identity was kept confidential. All research was reported in accordance with strengthening the reporting of observational studies in epidemiology (STROBE) statement^31^.

### Data collection and variables

Data collection, including the physical and biochemical measurements, took place during household visits by trained research assistants with health background. The World Health Organization (WHO) Stepwise Surveillance (STEPS) instrument was used for data collection^32^. This previously validated and publicly available tool was also used in Nepal’s only nationwide surveillance of non-communicable diseases risk factors^10^. Age, education, ethnicity, occupation, height and family income were obtained from COBIN Wave I survey where as other socio-demographic, behavioural and anthropometric information, as defined below were assessed.

#### Cardiometabolic risk factors

The outcome of interest was three well-known risk factors for CVD^33^, namely diabetes, hypertension, and overweight/obesity.

#### Hypertension

Three consecutive blood pressure measurements were taken using a digital blood pressure monitor after participants were seated for five minutes. The average of the last two measurements was recorded. Hypertension was defined as a systolic blood pressure of ≥140 mm Hg and/or diastolic blood pressure of ≥90 mm Hg, and/or self-reported anti-hypertensive therapy^34^.

#### Diabetes

Fasting blood glucose level was measured by the capillary finger prick method using a standardized digital glucometer. The fasting procedure is described elsewhere^9^. Diabetes was defined following the WHO’s standard definition, as blood glucose level ≥126 mg/dl (7.0 mmol/l) or currently on medication for raised blood glucose^35^. Additionally, participants’ family history of diabetes and history of heart disease were asked as a binary response.

#### Overweight/obesity

Anthropometric measurements for calculating body mass index (BMI) included height measured in meter (m) using a portable standard stature scales, and weight in kilogram (kg) using digital personal scales. Participant’s BMI calculated as weight/height^2^, was categorized into normal vs. overweight or obese (BMI ≥ 25 kg/m^2^)^36^.

#### Clustering of cardiometabolic risk factors

In line with our study objective, clustering of risk factors of the metabolic syndrome was an additional outcome of interest. Based on the three risk factors, participants could be categorized into two broad combinations of risk factors, i.e., those with two risk factors (six unique combinations of having either or both of the two conditions such as hypertension and diabetes; hypertension or diabetes; hypertension and overweight/obesity; hypertension or overweight/obesity; diabetes and overweight/obesity; diabetes or overweight/obesity), and those with three risk factors (two unique combinations: all three risk factors and at least one risk factor).

#### Behavioral risk factors

Behavioral risk factors included current smoking, harmful alcohol use, physical inactivity, and insufficient fruits and vegetables intake, were obtained by self-report. We used the STEPS survey tool, previously validated in Nepalese context, to measure current smoking in our study^32^. Current smoking was defined as smoking at least one cigarette per day for the past 12 months. Both ex-smokers (those who smoked previously but quit subsequently) and those who never smoked cigarettes were categorized as current non-smokers. Participants were asked about the number of standard drinks consumed in the past 30 days. Among the current alcohol consumers (consumed alcohol in the past 30 days), consuming, in a single occasion per week, 8 or more standard drinks for females and 15 or more standard drinks for males was considered harmful alcohol use^37^. The Global Physical Activity Questionnaire (GPAQ)^38^ was used to assess the physical activity level from participants’ self-reported number of days and minutes spent on different moderate and/or vigorous activities for work, travel, and recreation. Low physical activity was defined as less than 3,000 metabolic equivalents tasks (MET) minutes of vigorous or moderate activity per week^39^. Participants reported consumption of number of servings of fruits and vegetables per week, later categorized ≥5 serving per week or <5 servings per week, following the recommended guidelines^40^.

### Socio-demographic factors

Age group (25–34, 35–44, 45–54, 55–64 years), sex (male/female), ethnicity (Upper caste/ Janajatis/ Dalits and ethnic minorities), and family’s monthly income (<20,000 and ≥20,000 NPR) were self-reported by the participants.

### Statistical analyses

All statistical analyses were performed using Stata 14.2 (StataCorp, College Station, Texas, USA). Characteristics of the study participants are expressed as mean and standard deviation (SD) for normal continuous variables, and as proportions for categorical variables. The socio-demographic and behavioral characteristics of the study participants across the risk factors and their clustering were compared by Pearson’s chi-squared test. Univariate and multivariate binary logistic regression analyses were performed to assess the covariates associated with cardiometabolic risk factors, individually and as clusters. The adjusted model included all the covariates included in this study, i.e., age group, sex, ethnicity, monthly income, smoking, physical activity, fruits/vegetables intake, harmful alcohol use, family history of diabetes, and history of heart diseases, which were selected a priori.

### Ethical approval and informed consent

Ethical Review Board of Nepal Health Research Council approved this study (reference number 766; reg. no 263/2016). Respondents provided informed written consent before data collection was initiated. Participation was voluntary, and participants’ identity was kept confidential. All research was reported in accordance with strengthening the reporting of observational studies in epidemiology (STROBE) statement^9,31^.

## Supplementary information

Supplemetal Tables 1–2

## Data Availability

Data are available upon reasonable request made in writing to the study team.
