# Supplementary material for: The burden and correlates of multiple cardiometabolic risk factors in a semi-urban population of Nepal: a community-based cross-sectional study
Source: Sci Rep. 2019 Oct 25;9:15382. doi: 10.1038/s41598-019-51454-9 (PMC6814741; doi:10.1038/s41598-019-51454-9)
Supplement: Supplementary file 1 — Supplemetal Tables 1–2 [file 41598_2019_51454_MOESM1_ESM.docx]

**Supplementary Appendix**

**The burden and correlates of multiple cardiometabolic risk factors in a semi-urban population of Nepal: a community-based cross-sectional study**

Bishal Gyawali^1,2^*, Shiva Raj Mishra^3^, Saruna Ghimire^4^, Martin Rune Hassan Hansen^5,6^, Kishor Jung Shah^1^, Koshal Subedee^1^, Pabitra Babu Soti^3^, Dinesh Neupane^7^, Per Kallestrup^5^

^1^Community Health Development Nepal (CHEDEN), Kathmandu, Nepal; ^2^Global Health Section, Department of Public Health, University of Copenhagen, Copenhagen, Denmark; ^3^Nepal Development Society, Bharatpur, Nepal; ^4^Department of Sociology and Gerontology, Miami University, Oxford, OH, United States of America; ^5^Department of Public Health, Aarhus University, Aarhus, Denmark; ^6^National Research Centre for the Working Environment, Copenhagen, Denmark; ^7^Department of Epidemiology, Welch Center for Prevention, Epidemiology, and Clinical Research Johns Hopkins Bloomberg School of Public Health, Baltimore, United States of America

**Supplemental Table**[**1**](https://www.nature.com/articles/s41598-018-27377-2#MOESM1)**. Distribution of cardiometabolic risk factors by demographic and behavioural characteristics**

| **Demographic and behavioural characteristics** | **Cardiometabolic risk factors n (%)** | | | **Two-way clustering of cardiometabolic risk factors n (%)** | | | | | | **Three-way clustering n (%)** | |
| --- | --- | --- | --- | --- | --- | --- | --- | --- | --- | --- | --- |
|  | **Diabetes (DM) 271 (11.7)** | **Hypertension (HTN)**  **797 (34.5)** | **Overweight/ obesity**  **1222 (52.9)** | **DM or HTN**  **911 (39.4)** | **Overweight/ obese**  **or HTN**  **1525 (66.0)** | **Overweight/ obese**  **or DM**  **1321 (57.2)** | **DM and HTN**  **157 (6.8)** | **Overweight/obese**  **and HTN**  **494 (21.4)** | **Overweight/ obese**  **and DM**  **172 (7.4)** | **Overweight/ obese**  **or DM or HTN**  **1575 (68.2)** | **Overweight/ obese**  **and DM and HTN**  **108 (4.7)** |
| **Age group** |  |  |  |  |  |  |  |  |  |  |  |
| 25-34 | 11 (4) | 44 (6) | 152 (12) | 53 (6) | 164 (11) | 158 (12) | 2 (1) | 32 (6) | 5 (3) | 169 (11) | 1 (1) |
| 35-44 | 49 (18) | 160 (20) | 402 (33) | 191 (21) | 451 (30) | 423 (32) | 18 (12) | 111 (22) | 28 (16) | 464 (29) | 10 (9) |
| 45-54 | 98 (36) | 273 (34) | 388 (32) | 316 (35) | 485 (32) | 419 (32) | 55 (35) | 176 (36) | 67 (39) | 501 (32) | 40 (37) |
| 55-64 | 113 (42) | 320 (40) | 280 (23) | 351 (39) | 425 (28) | 321 (24) | 82 (52) | 175 (36) | 72 (42) | 441 (28) | 57 (53) |
| **Sex** |  |  |  |  |  |  |  |  |  |  |  |
| Male | 113 (42) | 336 (42) | 321 (26) | 375 (41) | 474 (31) | 364 (28) | 74 (47) | 183 (37) | 70 (41) | 494 (31) | 51 (47) |
| Female | 158 (58) | 461 (58) | 901 (74) | 536 (59) | 1051 (69) | 957 (72) | 83 (53) | 311 (63) | 102 (59) | 1081 (69) | 57 (53) |
| **Ethnicity** |  |  |  |  |  |  |  |  |  |  |  |
| Upper caste | 127 (47) | 382 (48) | 612 (50) | 442 (49) | 776 (51) | 669 (51) | 67 (43) | 218 (44) | 70 (41) | 805 (51) | 39 (36) |
| Janjatis | 11 (41) | 290 (36) | 478 (39) | 327 (36) | 555 (36) | 509 (39) | 74 (47) | 213 (43) | 80 (47) | 568 (36) | 56 (52) |
| Dalit/ethnic minorities | 33 (12) | 125 (16) | 132 (11) | 142 (16) | 194 (13) | 143 (11) | 16 (10) | 63 (13) | 22 (13) | 202 (13) | 13 (12) |
| **Monthly income (NPR)** |  |  |  |  |  |  |  |  |  |  |  |
| <20000 | 90 (33) | 299 (38) | 383 (31) | 341 (37) | 510 (33) | 421 (32) | 48 (31) | 172 (35) | 52 (30) | 533 (34) | 33 (31) |
| ≥20000 | 181 (67) | 498 (62) | 839 (69) | 570 (63) | 1015 (67) | 900 (68) | 109 (69) | 322 (65) | 120 (70) | 1042 (66) | 75 (69) |
| **Current smoking** |  |  |  |  |  |  |  |  |  |  |  |
| Yes | 33 (12) | 151 (19) | 118 (10) | 165 (18) | 207 (13) | 134 (10) | 19 (12) | 62 (13) | 17 (10) | 214 (14) | 10 (9) |
| No | 238 (88) | 646 (81) | 1104 (90) | 746 (82) | 1318 (86) | 1187 (90) | 138818) | 432 (87) | 155 (90) | 1361 (86) | 98 (91) |
| **Physical activity** |  |  |  |  |  |  |  |  |  |  |  |
| Low | 50 (18) | 110 (14) | 140 (11) | 127 (14) | 185 (12) | 162 (12) | 33 (21) | 65 (13) | 28 (16) | 193 (12) | 19 (18) |
| High | 221 (82) | 687 (86) | 1082 (89) | 784 (86) | 1340 (88) | 1159 (88) | 124 (79) | 429 (87) | 144 (84) | 1382 (88) | 89 (82) |
| **Servings of fruits and vegetables** |  |  |  |  |  |  |  |  |  |  |  |
| ≥ 5 servings | 9 (3) | 44 (6) | 58 (5) | 49 (5) | 74 (5) | 61 (5) | 4 (3) | 28 (6) | 6 (3) | 76 (5) | 3 (3) |
| < 5 servings | 262 (97) | 753 (94) | 1164 (95) | 862 (95) | 1451 (95) | 1260 (95) | 153 (97) | 466 (94) | 166 (97) | 1499 (95) | 105 (97) |
| **Harmful alcohol use** |  |  |  |  |  |  |  |  |  |  |  |
| Yes | 36 (13) | 625 (78) | 128 (10) | 180 (20) | 212 (14) | 143 (11) | 28 (18) | 88 (18) | 21 (12) | 216 (14) | 17 (16) |
| No | 235 (87) | 172 (22) | 1094 (90) | 731 (80) | 1313 (86) | 1178 (90) | 129 (81) | 406 (82) | 151 (88) | 1359 (86) | 91 (84) |
| **Family history of diabetes** |  |  |  |  |  |  |  |  |  |  |  |
| Yes | 107 (39) | 178 (22) | 288 (24) | 228 (25) | 341 (22) | 320 (24) | 57 (36) | 125 (25) | 75 (44) | 359 (23) | 43 (40) |
| No | 164 (61) | 619 (78) | 934 (76) | 683 (75) | 1184 (78) | 1001 (76) | 129 (81) | 369 (75) | 97 (56) | 1216 (77) | 65 (60) |
| **History of heart diseases** |  |  |  |  |  |  |  |  |  |  |  |
| Yes | 22 (8) | 45 (6) | 38 (3) | 50 (5) | 56 (4) | 50 (4) | 17 (11) | 27 (5) | 10 (6) | 60 (4) | 9 (8) |
| No | 249 (92) | 752 (94) | 1184 (97) | 861 (95) | 1469 (96) | 1271 (96) | 140 (89) | 467 (95) | 162 (94) | 1515 (96) | 99 (92) |

**Supplemental Table**[**2.**](https://www.nature.com/articles/s41598-018-27377-2#MOESM1) **Two-way clustering of cardio-metabolic risk factors by demographic and behavioral characteristics**

| **Demographic and behavioral characteristics** | **Hypertension and diabetes** | **Hypertension or diabetes** | **Hypertension and overweight/obesity** | **Hypertension or overweight/obesity** | **Diabetes and overweight/obesity** | **Diabetes or overweight/obesity** |
| --- | --- | --- | --- | --- | --- | --- |
|  | **OR (95% CI)** | **OR (95% CI)** | **OR (95% CI)** | **OR (95% CI)** | **OR (95% CI)** | **OR (95% CI)** |
|  | **Unadjusted** | **Unadjusted** | **Unadjusted** | **Unadjusted** | **Unadjusted** | **Unadjusted** |
| **Age group** |  |  |  |  |  |  |
| 25-34 | **Ref** | **Ref** | **Ref** | **Ref** | **Ref** | **Ref** |
| 35-44 | 4.36 (1.00-18.95) | 1.75 (1.24-2.46) | 1.91 (1.22-2.00) | 1.52 (1.14-2.01) | 2.87 (1.09-7.63) | 1.38 (1.04-1.82) |
| 45-54 | 15.72 (3.80-65.05) | 3.41 (2.45-4.75) | 2.82 (1.83-4.35) | 1.52 (1.15-2.00) | 5.66 (2.23-14.36) | 1.12 (0.85-1.47 |
| 55-64 | 35.95 (8.75-147.79) | 5.81 (4.14-8.14) | 3.50 (2.25-5.42) | 1.66 (1.24-2.21) | 6.28 (2.48-15.91) | 0.89 (0.67-1.17) |
| **Sex** |  |  |  |  |  |  |
| Male | **Ref** | **Ref** | **Ref** | **Ref** | **Ref** | **Ref** |
| Female | 0.39 (0.28-0.55) | 0.50 (0.42-0.59) | 0.85 (0.67-1.07) | 1.11 (0.92-1.33) | 0.88 (0.63-1.22) | 1.59 (1.33-1.89) |
| **Ethnicity** |  |  |  |  |  |  |
| Upper caste | **Ref** | **Ref** | **Ref** | **Ref** | **Ref** | **Ref** |
| Janjatis | 2.16 (1.52-3.07) | 1.45 (1.20-1.74) | 2.50 (1.94-3.22 | 1.83 (1.50-2.24) | 2.87 (2.01-4.09) | 1.91 (1.58-2.31) |
| Dalit/ ethnic minorities | 1.13 (0.64-2.00) | 1.52 (1.18-1.95) | 1.15 (0.82-1.62) | 1.00 (0.77-1.28) | 1.08 (0.65-1.79) | 0.73 (0.57-0.94) |
| **Monthly income (NPR)** |  |  |  |  |  |  |
| <20000 | **Ref** | **Ref** | **Ref** | **Ref** | **Ref** | **Ref** |
| ≥20000 | 1.17 (0.82-1.67) | 0.86 (0.72-1.03) | 1.20 (0.95-1.52) | 1.28 (1.07-1.53) | 1.54 (1.08-2.19) | 1.43 (1.20-1.70) |
| **Current smoking** |  |  |  |  |  |  |
| Yes | **Ref** | **Ref** | **Ref** | **Ref** | **Ref** | **Ref** |
| No | 1.21 (0.73-2.00) | 0.75 (0.60-0.94) | 1.76 (1.28-2.41) | 1.60 (1.28-2.02) | 2.78 (1.65-4.68) | 2.70 (2.14-3.40) |
| **Physical activity** |  |  |  |  |  |  |
| Low | **Ref** | **Ref** |  | **Ref** |  |  |
| High | 1.21 (0.73-2.00) | 0.67 (0.52-0.87) | 0.74 (0.52-1.05) | 0.81 (0.61-1.07) | 0.59 (0.38-0.93) | 0.82 (0.63-1.06) |
| **Servings of fruits and vegetables** |  |  |  |  |  |  |
| ≥ 5 servings | **Ref** | **Ref** | **Ref** | **Ref** | **Ref** | **Ref** |
| < 5 servings | 2.11 (0.76-5.84) | 0.97 (0.67-1.40) | 1.08 (0.67-1.75) | 1.28 (0.88-1.86) | 1.82 (0.77 (4.27) | 1.36 (0.94-1.96) |
| **Harmful alcohol use** |  |  |  |  |  |  |
| No | **Ref** | **Ref** | **Ref** | **Ref** | **Ref** | **Ref** |
| Yes | 2.17 (1.39-3.40) | 2.47 (1.93-3.15) | 1.57 (1.15-2.16) | 1.17 (0.91-1.52) | 0.70 (0.43-1.14) | 0.61 (0.48-0.78) |
| **Family history of diabetes** |  |  |  |  |  |  |
| No | **Ref** | **Ref** | **Ref** | **Ref** | **Ref** | **Ref** |
| Yes | 2.94 (2.06-4.19) | 1.72 (1.40-2.11) | 1.99 (1.50-2.64) | 1.53 (0.90-2.60) | 4.89 (3.44-6.95) | 2.02 (1.62-2.52) |
| **History of heart diseases** |  |  |  |  |  |  |
| No | **Ref** | **Ref** | **Ref** | **Ref** | **Ref** | **Ref** |
| Yes | 6.67 (3.51-12.65) | 3.19 (1.95-5.19) | 2.33 (1.28-4.23) | 1.53 (0.90-2.60) | 2.38 (1.12-5.04) | 1.51 (0.93-2.46) |
